# Editorial

**Published:** 1849-04

**Authors:** 


THE MEDICAL EXAMINER.

PHILADELPHIA, APRIL, 1849.

### NEW ORLEANS MEDICAL AND SURGICAL JOURNAL.

By the January No. of this valuable periodical, we learn that Dr. J. Harrison has withdrawn from the editorial department in consequence of professional and other engagements, and that the whole labor now falls upon Dr. Hester. To Dr. Harrison it must be a source of congratulation that he is released from the onerous duties of an editor; and while we regret the withdrawal of his able hand from the editorial corps, we sincerely trust that Dr. Hester, who now conducts the Journal alone, may have "his back strengthened to the burthen."

Since writing the above, we regret to learn the death of Dr. Harrison. His loss will be severely felt by the University of Louisiana, in which he held the Professorship of Physiology and Pathology, and by his numerous friends.

### MEDICAL DEPARTMENT, U. S. ARMY.

The warm interest that we feel in the proper appreciation of the medical department, both of the Army and Navy, will, we trust, prove a sufficient apology for the insertion of the following circular from the Surgeon General, Dr. Lawson, which we have extracted from the Boston Medical and Surgical Journal:

*Surgeon General's Office, Feb.* 7, 1849.

Sir,—The position to which the Medical Staff of the Army has attained after a long struggle against prejudice and error, and in opposition to views entertained by some high in authority, is a gratifying illustration that "truth is powerful and will ultimately prevail."

The Officers of the Medical Department may now rest satisfied that their position in the Army cannot be successfully assailed by arguments addressed to the reason; and that if driven from the ground they now occupy, it can only be through the temporary triumph of prejudice and authority over truth, reason, and justice. Conscious of the soundness of their claims to military rank, the members of the Corps will hold themselves prepared to meet any issues which may arise between themselves and others in authority; and appealing to law and regulation, resolve "to ask for nothing but what is right, to submit to nothing that is wrong."

While it is confidently anticipated that the senior officers of the Department will be governed by a sound discretion, and always pursue the course best calculated to secure their rights, it is deemed expedient to call the attention of its junior members to some considerations which may aid their inexperience and lead them to a correct appreciation of their military position.

On the day of his appointment, an Assistant Surgeon is invested by law with the rank of First Lieutenant. This rank will give him precedence, (except where military command is implied,) of all Second Lieutenants, and of all First Lieutenants whose appointments as such date subsequent to his own commission.

On all details, then, for Courts Martial, Military Boards, and other mixed commissions where military command is not involved—each member acting independently, and giving his vote free from military control—the rank of the Medical officer will take effect; and against any order convening such Boards, &c., which embraces him in the detail without a recognition of his military position, it is his duty firmly yet respectfully to protest.

It is enjoined upon all Medical officers to take, in a conciliatory spirit, a decided stand upon this point at the very outset; and for the reason that encroachment, promptly met, will be more promptly checked; while any evidence of irresolution, or want of confidence in the correctness of their position, might lead to further aggression.

The time is propitious for the acknowledgment of the claims of the Medical Staff to a specific military position. During the war with Mexico, their conduct in the field was the subject of high commendation; and there is a current of feeling in their favor which, if not diverted from its course by acts of indiscretion on the part of the officers themselves, will go far to remove the prejudices which have hitherto opposed the recognition of their rights.

---

BOARD OF EXAMINERS, U. S. ARMY.

Among the acts passed by the last Congress, was one to increase the Medical Staff of the Army, and it is now officially announced that a Board of Medical Examiners, consisting of Surgeons Mower, Wood, and Cuyler, and Assistant Surgeon Thomas Henderson, will convene in New York on the 1st of May, for the examination of Assistant Surgeons for promotion, and such applicants for appointment in the Medical Staff of the Army as may be invited to attend. The junior member acts as recorder. The sessions of the Board will be continued during a month, at least. Candidates being between the ages of 21 and 28 years, should forward their applications to the Secretary of War, accompanied by respectable testimonials of moral character and physical qualifications. There are nine vacancies existing in the medical department in the grade of Assistant Surgeons.

---

NATIONAL MEDICAL ASSOCIATION.

The Committee of Arrangements, for the next Annual Meeting of the National Medical Association, hereby invite all Medical Societies and Institutions throughout the United States, to send delegates, according to the rules presented in the Constitution, viz :—

"The delegates shall receive their appointments from permanently organized Medical Societies, Medical Colleges, Hospitals, Lunatic Asylums, and other permanently organized Medical Institutions, of good standing in the United States. Each delegate shall hold his appointment for one year, or until another is appointed to succeed him, and shall participate in all the business affairs of the Association.

"Each local Society shall have the privilege of sending to the Association one delegate for every ten of its regular resident members, and one for every additional fraction of more than half of this number. The faculty of every regularly constituted Medical College, or chartered School of Medicine, shall have the privilege of sending two delegates. The professional staff of every chartered, or municipal Hospital, containing an hundred inmates or more, shall have the privilege of sending two delegates; and every other permanently organized medical institution, of good standing, shall have the privilege of sending one delegate."

The meeting will be held in Boston, on the first Tuesday in May, and Societies are requested to forward lists of the names of their delegates to the Secretary, Dr. Henry I. Bowditch, so that the documents may reach Boston on or before April 10th.

The Committee would likewise give notice that, according to the vote passed at the last meeting of the Association, they will be in session on the day previous to the annual meeting, and they earnestly invite all permanent members and delegates, who arrive on or before that day, to enrol their names then, so that no time may be lost to the Association on the first day of the meeting.

JACOB BIGELOW, *Chairman.*

---

MEDICAL SOCIETY OF THE STATE OF PENNSYLVANIA.

The annual meeting of the Medical Society of the State of Pennsylvania will be held in the City of Reading, on Wednesday, April 11th, 1849. The officers of the County Societies are requested to forward the credentials of their delegates to the undersigned.

HENRY S. PATTERSON, M. D.,
GEORGE B. KERFOOT, M. D.,
*Recording Secretaries.*

